# Controle da Frequência Cardíaca na Insuficiência Cardíaca

**DOI:** 10.36660/abc.20200572

**Published:** 2020-12-01

**Authors:** Mariana Janini Gomes, Luana Urbano Pagan

**Affiliations:** 1 Brigham and Women’s Hospital Massachusetts EUA Brigham and Women’s Hospital, Massachusetts - EUA; 2 Universidade Estadual Paulista Júlio de Mesquita Filho Faculdade de Medicina BotucatuSP Brasil Universidade Estadual Paulista Júlio de Mesquita Filho - Campus de Botucatu Faculdade de Medicina, Botucatu, SP – Brasil

**Keywords:** Insuficiência Cardíaca/tratamento, Frequência Cardíaca, Equipe de Assistência do Paciente, Adesão a Medicação

A insuficiência cardíaca (IC) constitui problema clínico de extrema importância devido à sua alta prevalência e à gravidade das manifestações clínicas.^1 , 2^ O aumento da longevidade da população tem elevado o número de hospitalizações e óbitos por IC. Últimos dados divulgados pelo DATASUS (2020) mostram que ocorrem 26.482 mortes por IC.^3^

A característica multifatorial da síndrome exige que a escolha do tratamento seja realizada por equipe multiprofissional e atenta para as recomendações propostas nas diretrizes de tratamento da IC. O acompanhamento e monitorização dos pacientes são essenciais para a melhora dos desfechos clínicos.^4 - 6^

O controle da frequência cardíaca (FC) é considerado alvo terapêutico no tratamento da IC, visto que FC elevada é marcador de eventos em IC.^4^ Diversos estudos evidenciaram a importância do controle da FC para melhorar os desfechos como risco de morte e admissão hospitalar por IC.^7 , 8^

Portanto, estudos observacionais que monitoram pacientes com IC são fundamentais para apresentar um panorama da efetividade dos tratamentos na IC. Nesse sentido, estudo realizado por Cardoso et al.^9^ teve o objetivo de avaliar se os pacientes acompanhados em ambulatório de cardiologia tem sua FC controlada e recebem prescrição de medicação para IC adequada segundo as diretrizes.^9^

Em um recente estudo global foi observado que, apesar de satisfatória aderência às recomendações das diretrizes de tratamento da IC, é observada subdosagem dos medicamentos recomendados. São necessárias ações para melhorar essa situação, pois altas doses das terapias recomendadas são associadas à redução da mortalidade em estudos observacionais de IC.^10^

Em seu estudo, Cardoso et al.^9^ ressaltam que a redução da FC com os betabloqueadores não é igual para todos os pacientes. Levantando, dessa forma, o importante questionamento do que seria mais importante para determinar uma boa evolução dos pacientes com IC, a dose alvo do betabloqueador ou a redução da FC?

O estudo avaliou 171 pacientes e mostraram dados muito relevantes sobre a eficácia do tratamento da IC sobre o controle da FC. Apesar de o betabloqueador ter sido prescrito em doses elevadas para 98,8% dos pacientes, uma grande parcela (40,9%) apresentou FC maior que 70 batimentos por minuto. Os autores observaram também que outras classes de medicamentos recomendados para tratamento da IC, como inibidores da enzima conversora de angiotensina, bloqueadores do receptor da angiotensina e diuréticos, estavam sendo prescritos adequadamente.^9^

Dessa forma, considerando o impacto da FC no prognóstico de pacientes com IC, o estudo conduzido por Cardoso et al.^9^ sugere que outras medidas precisam ser adotadas, além da prescrição das doses alvo dos medicamentos indicados pelas diretrizes, para manter o controle da FC em pacientes com IC.
