# Supplementary material for: Controle da Frequência Cardíaca na Insuficiência Cardíaca
Source: Arq Bras Cardiol. 2020 Dec 1;115(6):1070–1. [Article in Portuguese] doi: 10.36660/abc.20200572 (PMC8133739; doi:10.36660/abc.20200572)
Supplement: Translation

# Translation: Heart Rate Control in Heart Failure

**Authors:** Mariana Janini Gomes, Luana Urbano Pagan

**Affiliations:** 1 USA Brigham and Women’s Hospital, Massachusetts - USA; 2 Brazil Universidade Estadual Paulista Júlio de Mesquita Filho - Campus de Botucatu Faculdade de Medicina, Botucatu, SP – Brazil

**Keywords:** Heart Failure/treatment, Heart Rate, Patient Care Team, Medication Adherence

Heart failure (HF) is an important public health issue due to its high prevalence and the severity of clinical manifestations.^1 , 2^ The increment in population longevity has increased the number of hospitalizations and deaths due to HF. Recent data by DATASUS (2020) show the occurrence of 26,482 deaths from HF.^3^

This syndrome has a multifactorial characteristic and its treatment requires a multidisciplinary team to meet the recommendations proposed in the guidelines for the treatment of HF. Follow-up and monitoring of patients are essential for improving the clinical outcomes.^4 - 6^

Heart rate (HR) is considered a therapeutic target in the treatment of HF, since elevated HR is a marker of events in HF.^4^ Several studies have shown the importance of HR control to improve outcomes such as risk of death and hospital admission by HF.^7 , 8^

Therefore, observational studies are essential to investigate the impact of the treatment of patients with HF. In this sense, a study carried out by Cardoso et al.^9^ aimed to assess whether patients monitored in outpatient settings have their HR controlled and receive drug prescriptions according to the guidelines for the treatment of HF.^9^

A recent global study showed that despite satisfactory adherence to the recommendations of guidelines for the treatment of HF, an under-dosage of the recommended drugs is observed. Actions are needed to improve this situation, since high doses of recommended therapies are associated with reduced mortality in observational studies of patients with HF.^10^

In their study, Cardoso et al.^9^ mentioned that beta-blockers do not have the same effect reducing HR for all patients. Thus, the authors raised an important question of what would be more important to determine a good outcome for patients with HF, the target dose of the beta-blocker or the reduction in HR?

The study evaluated 171 patients in outpatient settings and showed very relevant data on the effectiveness of the treatment of HF on HR control. Although beta-blockers were prescribed in high doses for 98.8% of patients, a large portion (40.9%) had HR greater than 70 beats per minute. The authors also observed that other classes of drugs recommended for the treatment of HF, such as angiotensin-converting enzyme inhibitors, angiotensin receptor blockers and diuretics, were properly prescribed.^9^

Thus, considering the impact of HR on the prognosis of patients with HF, the study conducted by Cardoso et al.^9^ suggests that other actions in addition to the prescription of the recommended drug therapy need to be adopted to ensure the control of HR in patients with HF.
